# Genomic newborn screening: public health policy considerations and recommendations

**DOI:** 10.1186/s12920-017-0247-4

**Published:** 2017-02-21

**Authors:** Jan M. Friedman, Martina C. Cornel, Aaron J. Goldenberg, Karla J. Lister, Karine Sénécal, Danya F. Vears, the Global Alliance for Genomics and Health Regulatory and Ethics Working Group Paediatric Task Team, Jan M. Friedman, Martina C. Cornel, Khalid Al-Thihli, Pascal Borry, David Flannery, Aaron Goldenberg, Anne Junker, Stephen Kingsmore, Nigel G. Laing, Erick Scott, Ambroise Wonkam, Bartha Knoppers

**Affiliations:** 1 Department of Medical Genetics, University of British Columbia, Vancouver, Canada; 2 Child & Family Research Institute, Vancouver, Canada; 3 Section Clinical Genetics, Department of Clinical Genetics, VU University Medical Center, Amsterdam, Holland; 4 EMGO Institute for Health and Care Research, VU University Medical Center, Amsterdam, Holland; 5 The Center for Genetic Research Ethics and Law, Department of Bioethics, Case Western Reserve University, Cleveland, OH USA; 6 Office of Population Health Genomics, Public Health Division, Department of Health, Government of Western Australia, Perth, Australia; 7 Centre of Genomics and Policy, Department of Human Genetics, McGill University, Montreal, Canada; 8 Centre for Biomedical Ethics and Law, Department of Public Health and Primary Care, KU Leuven, Leuven, Belgium

**Keywords:** Newborn Screening, Whole Genome Sequencing, Exome Sequencing, Public Policy, Ethics, Public Health Genetics

## Abstract

**Background:**

The use of genome-wide (whole genome or exome) sequencing for population-based newborn screening presents an opportunity to detect and treat or prevent many more serious early-onset health conditions than is possible today.

**Methods:**

The Paediatric Task Team of the Global Alliance for Genomics and Health’s Regulatory and Ethics Working Group reviewed current understanding and concerns regarding the use of genomic technologies for population-based newborn screening and developed, by consensus, eight recommendations for clinicians, clinical laboratory scientists, and policy makers.

**Results:**

Before genome-wide sequencing can be implemented in newborn screening programs, its clinical utility and cost-effectiveness must be demonstrated, and the ability to distinguish disease-causing and benign variants of all genes screened must be established. In addition, each jurisdiction needs to resolve ethical and policy issues regarding the disclosure of incidental or secondary findings to families and ownership, appropriate storage and sharing of genomic data.

**Conclusion:**

The best interests of children should be the basis for all decisions regarding the implementation of genomic newborn screening.

## Background

The Global Alliance for Genomics and Health is an international collaboration of more than 400 healthcare, research, disease advocacy, life science, and information technology institutions working together to promote human health through sharing of genomic and clinical data [http://genomicsandhealth.org/]. Within this remit, the Paediatric Task Team of the Global Alliance’s Regulatory and Ethics Working Group [http://genomicsandhealth.org/working-groups/regulatory-and-ethics-working-group] was established to address issues of particular relevance to child health.

Recent research has demonstrated that genomic technology, and particularly genome-wide (whole genome or exome) sequencing, can identify genetic causes of rare paediatric diseases much more effectively than conventional clinical and laboratory methods [1, 2]. Furthermore, genome-wide sequencing could, at least in theory, be used in newborn screening to identify many more serious health conditions than is possible today [3–8]. This possibility interests some parents [9–11], commercial testing laboratories [12], and the US National Institutes of Health [http://www.nih.gov/news-events/news-releases/nih-program-explores-use-genomic-sequencing-newborn-healthcare], but it also raises serious ethical and public policy concerns [3–5, 13–20].

## Methods

The Global Alliance Paediatric Task Team developed the recommendations shown in Table 1 for clinicians, clinical laboratory scientists, and policy makers regarding our current understanding, concerns and consensus regarding the use of genomic technologies for population-based newborn screening. This document and its recommendations were reviewed and approved by the Paediatric Task Team in December 2015. These recommendations should be reconsidered in the future as our knowledge in these areas improves.

**Table 1:** Recommendations

1. Newborn screening by any method, including genomic testing, if adopted as a public health program should be equally available and accessible to every infant born in the jurisdiction. 2. Interpretation of genomic newborn screening results requires extensive knowledge of the normal (benign) variants, as well as of pathogenic variants, of every gene tested. Genomic newborn screening programs should, therefore, make population-specific allele frequencies of every gene included in the program publicly available in a freely-accessible database. The functional consequences (benign, pathogenic, or undetermined) of each allele should also be made available, along with the evidence supporting functional interpretations. 3. Publicly-funded universal newborn screening by genomic methods should be limited to diseases that can be diagnosed in the newborn period and effectively treated or prevented in childhood. 4. If population-based genomic newborn screening is introduced, it should only be offered as part of a comprehensive public health program that includes appropriate confirmatory testing, therapeutic interventions, clinical follow-up, genetic counselling, quality assurance, public and professional education, and governance and oversight. 5. Newborn screening by next-generation sequencing or other genomic methods should only be considered as an add-on to current first tier screening programs. 6. Current newborn screening should *not* be replaced by next generation sequencing or other genomic methods for any disease unless the genomic technology has been shown to have equal or better sensitivity and specificity for the disease. 7. At the present time, our understanding of, and ability to interpret genomic variants does not justify use of genome-wide (whole genome or exome) sequencing in population-based newborn screening. Research is needed to demonstrate the clinical utility and cost-effectiveness of genome-wide sequencing and to resolve outstanding health policy and ethical issues before genome-wide sequencing is implemented for newborn screening within a jurisdiction. 8. At the present time, our understanding of, and ability to interpret genomic variants does not justify sequencing large multigene (physical or bioinformatic) panels for population-based newborn screening. Research is needed to demonstrate the clinical utility and cost-effectiveness of sequencing large multigene panels for population-based newborn screening and to resolve outstanding health policy and ethical issues before the use of large sequencing panels is implemented for newborn screening within a jurisdiction.	

Following a brief overview of current programs and public health policies regarding newborn screening by other means, we describe the genomic technologies that could be used for newborn screening and discuss how genomic newborn screening might differ from conventional newborn screening. We then consider issues of concern related to genomic newborn screening and provide justification for each of our recommendations. We conclude by considering the public health opportunity genomic newborn screening offers and highlight the need for more research in this area.

## Results

### Newborn screening today

Newborn screening is the process by which infants are tested for conditions that can cause death, serious life-long disability or chronic disease if not treated shortly after birth. The purpose of newborn screening is to identify conditions for which effective therapy is available and to provide this treatment early enough to prevent or ameliorate the disease, so that affected children can live healthier lives.

Newborn screening began in the early 1960s for inborn errors of metabolism such as phenylketonuria (PKU) and is now routinely performed for a variety of conditions on almost all infants in many countries. Given this history and wide acceptance, the essential elements of population-based newborn screening programs have become well understood. At their heart, they are organized approaches to early detection, through which asymptomatic individuals in a specific population are systematically tested for a set of conditions or for biomarkers of the conditions. The programs aim to identify these conditions at an early stage, generally prior to the onset of symptoms. Screening must usually be followed by a more definitive diagnostic process for the condition. Once a serious condition is identified in a newborn infant, treatment or management designed to ameliorate or prevent the onset of symptoms must be initiated.

Newborn screening programs vary greatly from jurisdiction to jurisdiction with respect to which conditions and how many diseases are tested for. Most newborn screening is performed on a small blood sample obtained by heel prick from each baby. Testing this sample by tandem mass spectrometry, a method of identifying and quantitating many metabolites simultaneously, permits recognition of about 50 potentially treatable inborn errors of metabolism, although most jurisdictions that do population-based newborn screening test for only a subset of these conditions.

Only genetic abnormalities that are associated with major alterations of biochemicals in the blood can be detected by tandem mass spectrometry, but other treatable conditions, such as congenital hypothyroidism, cystic fibrosis, sickle cell disease, and severe combined immunodeficiency, can be screened in the blood spot with other kinds of tests. A few additional disorders, such as congenital hearing loss and critical congenital heart disease, may be screened by methods that require physical measurements directly on the infant rather than analysis of a blood sample.

Newborn screening and early diagnosis of serious disease might seem to be advantageous under all circumstances. However, there are several countervailing factors that must also be considered. These include the implications of false positive and false negative screening results; of parental stress and anxiety about when, whether and how the disease will appear; of the often uncertain utility of available treatments; and of the social and personal cost of the entire program of screening and management of early or asymptomatic disease.

In 1968, under the auspices of the World Health Organization, Wilson and Jungner [21] developed criteria to assess the value of potential screening programs as public health interventions (Table 2). Almost 50 years later, the Wilson and Jungner criteria still provide a useful framework for assessing the value and appropriateness of newborn screening programs, although some of the criteria have been criticized and modifications proposed in light of more recent scientific developments and circumstances [22, 23].

**Table 2:** Wilson and Jungner [21] Criteria

1. The condition sought should be an important health problem. 2. There should be an accepted treatment for patients with recognized disease. 3. Facilities for diagnosis and treatment should be available. 4. There should be a recognizable latent or early symptomatic stage. 5. There should be a suitable test or examination. 6. The test should be acceptable to the population. 7. The natural history of the condition, including development from latent to declared disease, should be adequately understood. 8. There should be an agreed policy on whom to treat as patients. 9. The cost of case-finding (including diagnosis and treatment of patients diagnosed) should be economically balanced in relation to possible expenditure on medical care as a whole. 10. Case-finding should be a continuing process and not a “once and for all” project.	

Newborn screening programs follow defined protocols to produce population-level benefits through reductions in mortality and morbidity related to the conditions screened. The system generally includes most, if not all, of the following elements: Informing the family of the testing, obtaining (or presuming) their consent, obtaining the sample, performing the test, interpreting it, informing the child’s physician or parents of “screen-positive” (or “screen-negative”) results, arranging and performing confirmatory diagnostic testing, and initiating preventative management or treatment, when indicated. The successful delivery of a screening program is dependent upon efficient and timely activities at each stage, delivered in line with established policies, protocols, administration and governance. Additional components of a successful screening program are continuous quality management, monitoring and evaluation. These are necessary to demonstrate to funders, clinicians and the public that the program is achieving its objectives, justifying the continued investment of public resources.

### Ethical and public policy issues raised by current newborn screening practices

Population-based newborn screening with treatment or prevention of the serious conditions identified is one of the most successful public health interventions ever devised [24–26]. Almost every baby in most developed countries and many developing countries currently undergoes newborn screening for various serious early-onset diseases [27]. Introduction of genomic technology may provide an opportunity to identify more infants for whom early interventions can prevent serious illnesses, major handicaps or death. However, precautions must be taken to ensure that genomic technology is used in a manner that does not compromise the effectiveness or societal support of current screening programs. In order to be successful, genomic newborn screening must learn from the experience of conventional newborn screening over the past 50 years. These lessons are briefly reviewed here.

#### Consent for newborn screening

One of the most problematic issues in population-based newborn screening is whether parents should be asked for permission before testing takes place. While some programs do obtain explicit parental consent for newborn screening, most presume consent unless the parents express an objection. Such implicit consent is justified by the belief that newborn screening is in the child’s best interest. Newborn screening has been established as compulsory in some jurisdictions under a public health mandate, but even mandatory programs usually allow parents to “opt out” if they hold religious or other beliefs that are contrary to screening.

As programs have expanded to include conditions with wider phenotypic variability, unclear risk associations, and more invasive or less effective treatments, there are concerns that the justification for implicit consent or mandatory screening has been compromised [13, 28, 29]. Alternative suggestions include specific parent consent (i.e., “opting in”) for all newborn screening or a tiered approach in which some tests would require explicit parental consent while others would not. The tiered approach would be designed to maintain the benefits of universal screening for conditions where it is essential for the benefit of the child while allowing parents to choose whether or not to screen for conditions that do not meet the standards for compulsory population-wide screening. Unfortunately, however, a number of studies have found poor understanding of newborn screening among parents [13, 30–32]. This may prevent them from making informed choices about screening if options are made available. In addition, keeping track of and modifying the reporting in response to varying parental requests would greatly increase the administrative complexity and thus the cost of a newborn screening program.

#### What conditions should be screened for?

In accordance with the Wilson and Jungner criteria, newborn screening began in all jurisdictions with conditions that are life-threatening or could cause severe disability, are easy to screen for, and have an effective treatment. In their landmark paper supporting compulsory PKU screening, Faden, Holtzman and Chwalow [33] highlighted the harm that would likely occur in a newborn infant who was not screened and argued that this greatly outweighs the benefit of permitting parental choice about screening. As it became possible to screen for other conditions, similar criteria were required for additions to the screening panel. The association of disease severity and treatability in all of the conditions tested for provided the moral justification for making newborn screening mandatory in many jurisdictions.

While the Wilson and Jungner criteria have generally been used to assess the benefits and harms of adding conditions to the screening panel, programs may interpret these criteria differently and may also be subject to different political or public pressures to add certain conditions to the panel. As a consequence, different programs often screen for different conditions [34–36]. Some jurisdictions screen for fewer than ten conditions, and others screen for more than fifty.

The potential to screen for such a large number of conditions was made possible by the introduction of tandem mass spectrometry, which permits the addition of new screening targets to an existing metabolic panel at almost no cost by simply adjusting the analytical software. As a result, the kinds of conditions that have been proposed and added to newborn screening panels in some jurisdictions have begun to challenge conventional ethical norms. Some of the newly added conditions are not completely penetrant – not all infants who have the pathogenic biomarker develop the disease. In other instances, those who develop the disease may do so at a wide range of ages, from early childhood to adulthood, or may exhibit a wide range of disease severity or response to treatment. In such cases, some infants who screen positive may endure unnecessary diagnostic procedures and treatments, and their families may suffer increased stress and anxiety as they deal with future uncertainty. This could place a substantial burden on the health care system, with potentially negative effects [4]. These issues are amplified if the available treatment is expensive or associated with serious risks of adverse effects, as occurs with hematopoietic stem cell transplantation, for example.

#### The benefits of newborn screening

Concerns have also been raised about the kinds of benefits expanded newborn screening programs provide [37]. Treatment has traditionally been defined in terms of preventing the occurrence of symptoms related to a condition or substantially ameliorating those symptoms if they do occur. In recent years, however, the concept of “treatable” has been expanded to include reducing symptoms to some degree, prolonging life, or avoiding long diagnostic quests once symptoms appear [38]. Furthermore, some advocates have pointed out that interventions which have not been proven to be effective in reducing morbidity or mortality might, nevertheless, benefit some children or their families [23]. Families may also value the opportunity to participate in disease-related research.

Cystic fibrosis, which is now screened for in many North American, European and Australian jurisdictions, provides an excellent example. Infants with cystic fibrosis rarely die or suffer irreversible damage in the newborn period, but moderate clinical benefits have been demonstrated in children with cystic fibrosis who are identified by newborn screening and receive earlier dietary and respiratory management in comparison to children who are not diagnosed until they become symptomatic [39–41].

At its core, newborn screening is intended to benefit individual infants, but the justification for adding some new conditions to the screening panel has been to provide benefits beyond the infants to their families or society. For example, providing families information about an infant’s carrier status for a recessive genetic disorder such as cystic fibrosis or sickle cell disease, while of no immediate clinical benefit to the child, may allow the parents to make future reproductive choices that would not otherwise be available to them [42, 43]. Some have argued that reporting such findings should be avoided because doing so increases the cost of reporting and follow up in the screening program and has not been shown to be beneficial [42, 44]. Others advocate informing families of such results, which are produced incidentally by screening for primary targets with methods like tandem mass spectrometry or high-performance liquid chromatography of hemoglobin [45–47]. While expanding the scope of benefits considered may not be unethical, it does represent a shift in the goals of newborn screening that necessitates re-examination of its ethical justification.

#### Secondary use of newborn screening blood spots

Another issue that has raised controversy in several jurisdictions is retention and secondary use of leftover dried blood spots after newborn screening is complete. Residual blood spots are routinely used for internal laboratory quality assurance purposes and confirmation of original results [48]. In addition, the residual blood spots may be used to refine current methodologies and to develop new newborn screening tests [49]. These uses are generally accepted because they are related to the primary purpose of the blood collection [50, 51].

As blood spots are collected from almost all children at the time of birth, these samples also represent a unique population-based resource for biomedical research, public health surveillance, and forensic uses, such as the identification of disaster victims [52–55]. Biomedical and public health research using stored blood spots has contributed to our understanding in several important areas [56], including the development of childhood leukemia [57] and whether pregnant women are eating fish that contain excessive amounts of mercury [55]. However, the lack of consent from the parents for such uses is problematic, especially for programs that do not obtain consent for screening itself. As a consequence, policies regarding the retention and secondary use of newborn screening blood spots vary greatly worldwide [54, 55, 58–60] and secondary use of such blood spots has been the subject of several lawsuits in the United States and Canada [54].

### Genetic testing in newborn screening

#### Testing for mutations of individual genes and sets of genes

Many potentially treatable conditions cannot be detected in infants using current newborn screening methods [61]. Most of these disorders result from genetic mutations (either inherited from one or both of the parents or arising *de novo* in the child) and could, in principle, be diagnosed shortly after birth by means of available genomic technologies [4, 62, 63]. Examples include many early-onset seizure disorders, cardiac arrhythmias, cardiomyopathies, diseases of the blood or bone marrow, liver diseases and kidney disorders.

Clinical laboratories currently employ molecular genetic technologies for a variety of purposes, including the identification of bacteria or viruses involved in a patient’s infection and matching tissue antigens between a donor organ and a patient who requires organ transplantation. In addition, genetic testing is routinely performed by clinical labs in circumstances other than newborn screening – for example, to screen pregnant women for fetal Down syndrome [64–66] or in critically ill intensive care unit patients suspected of having a genetic disease. The latter approach has been successfully applied to newborn infants [67, 68], but it is important to distinguish this use of genome-wide sequencing for rapid diagnosis in a small number of critically ill infants from population-based newborn screening, where almost all babies, including those who are completely healthy, are tested [69].

There are several different kinds of genetic tests that could be used in newborn screening. Some employ conventional technologies; other tests are performed with massively-parallel (“next-generation”) sequencing machines, which, in comparison to the sequencers used in the Human Genome Project, produce 8,000,000 times more data 24,000 times faster at a cost that is 3,000,000 times lower [70–72]. Genetic tests include:

1. *Molecular genetic testing by methods that do not involve sequencing,* e.g., PCR of specific genetic targets. Such methods have been used in diagnostic testing for many years and are the clinical standard for rapid identification of infectious agents [73, 74]. A PCR-based technique has recently been adopted in some jurisdictions to screen newborn infants for severe combined immunodeficiency disease [75], a group of genetic disorders causing recurrent and eventually lethal infections that can be effectively treated by early stem cell transplantation. Simple molecular analytic technologies are also used to identify disease-causing germ-line mutations for confirmatory testing in some newborn screening programs [4] and even for primary screening in a few instances in which one or two specific mutations are responsible for almost all cases of a disease within a particular population. One example is newborn screening for glutaric acidemia type 1 caused by homozygosity for the *GCDH*, IVS1, G-T, +5 mutation in the Canadian province of Manitoba [76].
2. *Sequencing individual genes.* For more than 25 years, clinical laboratories have offered sequencing of individual genes, such as those for cystic fibrosis (*CFTR*) or Duchenne muscular dystrophy (*DMD*), to provide a molecular diagnosis in affected individuals. This testing is usually done by conventional (Sanger) sequencing of PCR-amplified coding regions of the gene. Individual gene sequencing is useful for clinical diagnosis in patients of any age, including newborn infants, but is too expensive and not sufficiently automatable to use for population-based screening. However, sequencing individual genes is used in some newborn screening programs for secondary or confirmatory testing of screen-positive infants [77–79].
3. *Gene panels.* Gene panels are sets of genes that are sequenced as a group. The group is selected because mutations of any of the included genes can produce clinically similar disease or, more broadly, diseases of the same class. The first panels offered for clinical diagnosis were small – three genes (*F8*, *F9* and *VWF*) for a coagulation disorder, for example – in essence just a few single gene tests done together. More recently, larger and larger gene panels have been developed, and it is now possible to obtain panels that test simultaneously for mutations in hundreds of genes associated with epilepsy or intellectual disability, or even for any of more than 3000 genes associated with mendelian diseases [80]. As the panels have grown larger, the technology employed has changed, with larger panels using higher through-put methods to capture the coding segments of the genes that are being tested, next generation methods for sequencing, and additional studies to look for mutations like genomic copy number changes that are difficult to identify by sequencing. Some laboratories offer “bioinformatic panels” that involve sequencing the coding regions of all genes (exome sequencing) but analyzing and reporting on only a selected subset of those genes.

#### Genome-wide (Whole genome or exome) sequencing

With the development of next-generation DNA sequencing technology and its substantial reduction in cost over recent years, sequencing all of the DNA (the *whole genome*) or the coding segments of all of the genes (the *exome*) in the cells of an individual all at once has emerged as a robust method of identifying mutations that cause treatment-resistant cancer [81, 82] or any of thousands of serious genetic conditions in patients with previously undiagnosed diseases [1, 83–88].

Most clinical laboratories currently utilize a different method, usually Sanger sequencing, to confirm pathogenic variants identified by genome-wide sequencing. However, clinical validity – whether recognizing disease-associated variants by sequencing (e.g., of the *CFTR* locus) predicts the disease (e.g., cystic fibrosis) – is often a more difficult question to resolve than analytical validity. No systematic studies of the clinical validity of genome-wide sequencing are available, but false positive and false negative reports of pathogenic variants are known to occur [1, 89, 90]. Such errors are more likely in circumstances like newborn screening, where the *a priori* chance that an individual will have any particular rare genetic disease is vanishingly small. Moreover, rigorous genotype-phenotype correlation, which is critical for clinical interpretation of genomic variants [91, 92], is impossible in most existing screening programs because information about illness or birth defects in the infants is not available to the screening laboratory.

#### Novel ethical and policy issues raised by genome-wide sequencing

All of the ethical and public policy issues associated with current newborn screening practices apply to genome-wide sequencing as well, and many of these issues are exacerbated by the fact that genome-wide sequencing produces much more information about the individual than conventional testing does. For example, it is more difficult (or impossible) to justify mandatory screening, even if families have the ability to opt out, if many additional screening targets are added, especially if the benefits of screening for some of these additional targets are uncertain. At the very least, genomic newborn screening would require ensuring that parents have sufficient, clearly-understandable information available about the screening program and that the entire population has access to confirmatory diagnostic and treatment services, including genetic counselling. Maintaining effective governance and efficient administration of population-based genomic newborn screening programs would also be essential to avoid losing the high participation rates and widespread public support that these programs currently enjoy.

##### Interpretation of genomic newborn screening results

The biggest challenge to using genome-wide sequencing to diagnose genetic disease is interpretation of the results [91, 93, 94]. The pathogenicity of genetic variants is often difficult to infer, especially if they are very rare or novel, as may often occur in general population screening. Rigorous criteria have been developed to define pathogenicity for clinical diagnostic labs, and the interpretation of variants is greatly aided by the accumulation of large databases of established pathogenic or benign variants [95, 96]. Nevertheless, some variants cannot be classified as either pathogenic or benign and must be reported as variants of uncertain significance (VUS), which can cause concern (often, but not always, unnecessarily) for individuals or families. In sick children who undergo diagnostic sequencing, the clinical phenotype can be used to help determine whether a variant is likely to be pathogenic by comparing the child’s phenotype to that expected if the variant were pathogenic. In contrast, the purpose of newborn screening is to identify infants with serious disorders *before* they become clinically apparent, and if the phenotype has not yet developed, it cannot be used to determine the pathogenicity of a genetic variant [1].

##### Return of genome-wide sequencing results

The uncertainty about interpretation raises questions regarding which variants laboratories should report back to clinicians and, in turn, to what extent there is an obligation to communicate these findings back to the patient [20, 97–100]. The resources required to investigate and communicate these findings may be a substantial burden on the health care system [3]. Other concerns include the potential for psychological harm to patients and their families, and the legal implications for laboratories and clinicians [101].

An additional complexity with genomic newborn screening relates to the fact that the testing is performed on infants who are legally incompetent when screened but who will gain competence when they grow older. This situation is not limited to newborn screening, of course – infants and young children are incompetent to make any decision regarding medical treatment or health screening. However, genomic newborn screening could detect diseases or predispositions to disease that do not have onset until middle or late adulthood, and we know that many adults choose *not* to have genetic testing for such conditions when it is offered [102–105]. Allowing substitute decision makers (usually the parents) to make decisions about such testing in infants raises issues relating to respect for future autonomy and privacy protection.

##### Genomic incidental findings

A major consideration if genome-wide (or large gene panel) sequencing were to be used for newborn screening is the frequent occurrence of “incidental findings” – genetic variants of potential importance to the child or family that are unrelated to the diseases for which the testing is performed [17, 106–110]. The use of the term “incidental” to describe these findings suggests that they are inadvertently found during the analysis of genomic data. This can and does happen, but it is also possible to look actively for genomic variants beyond those for which the screening is being performed. Variants of such secondary targets are sometimes called “secondary findings”. Others have used the terms “unsolicited”, “unanticipated”, or “adventitious” to describe incidental and/or secondary findings.

The frequency with which incidental or secondary findings are encountered can range from a few percent of patients to every single individual tested, depending on how the data are analyzed and what kinds of findings the testing laboratory reports. Even at the lowest frequency reported for genome-wide sequencing, incidental findings would be expected to occur more frequently than true positive results for disease-causing mutations associated with any of the rare genetic diseases tested for by conventional newborn screening.

Return of incidental findings that arise in diagnostic genome-wide sequencing is a contentious issue, and one that has elicited a number of sometimes-conflicting policy recommendations [4, 9, 17, 56, 97, 98, 101, 111, 112]. Areas of concern include the kinds of incidental or secondary findings that should be returned – should these only include “actionable” findings (i.e., those diagnostic of a condition for which an effective preventative or therapeutic intervention is available) or should findings that cannot be effectively prevented or treated also be returned? What about findings related to small or moderately increased risks of diseases that are common in the general population, or findings that may or may not be of value, depending on circumstances (e.g., pharmacogenetic variants or carrier status for recessive diseases)? Should patients be able to obtain results that are irrelevant medically but may have social importance (e.g., ancestry, potential for athletic performance, or genetic sex that differs from gender)?

Controversy has also arisen over which considerations should be prioritized with respect to the return of results. Should the focus be on returning any finding that could possibly be of benefit to an individual patient, or should incidental findings never be returned to maximize the cost-effectiveness of diagnosing serious diseases in the population as a whole? Should incidental findings only be returned if specifically requested by the patient (or their parents, if the patient is a child), or should such findings always be returned unless specifically declined? Return of information that is of no immediate benefit to a child but may be of benefit to other family members (e.g., the presence of pathogenic *BRCA1* mutation for hereditary breast and ovarian cancer in a little boy so that his mother can be tested for the mutation) is particularly contentious because it violates a core ethical principle that medical procedures in children are only justifiable if they directly benefit the child [101, 113].

In the context of population screening of infants, the ethical and policy concerns raised by return of incidental genomic findings are, if anything, even greater than for diagnostic genome-wide sequencing. Some have even questioned whether decisions about return of genomic information uncovered during newborn screening should be made by public health officials or policy-makers at all, arguing that all genomic data belong to the child, and that the parents, who are presumed to act most effectively in the child’s best interests, should decide what is of importance and what is not [114–117].

##### Storage of personal genomic data

As previously mentioned, storage and secondary use of infant blood spots obtained for population-based newborn screening is contentious, and similar issues arise for DNA samples isolated from these blood spots for genomic testing. Moreover, genome-wide sequencing would produce a large amount of information on each infant that is both potentially identifying and revealing of important medical or social issues. What should be done with these data once the newborn screening has been completed has generated substantial discussion. Some argue that a child could benefit from this information being stored in his or her electronic patient record for tailoring medical treatments to particular diseases that may arise later in life [3, 17]. These data would also provide very valuable research opportunities in areas such as population genetics, genome-wide association studies, penetrance of genetic disorders, and genotype-phenotype correlations [99].

The cost, risks and benefits of long-term storage of genomic data depend greatly on what is being stored and how it is stored. At one extreme, only the highest level results of newborn screening might be stored, e.g., “no cystic fibrosis-associated CFTR allele found”, while at the other extreme a complete list of all variants or each individual’s entire exome or whole genome sequence might be stored. The former does not differ from the storage of any other medical result in a health record, while the latter provides the greatest amount of both potentially beneficial and potentially harmful information. Moreover, storage of raw genome sequence would be of very little value without the ability to extract useful clinical information from it as needed and to return this information to the patient, family or physician in an appropriate manner. The cost of doing this is likely to remain far greater than the cost of data storage for the foreseeable future [16].

Others argue that the cost of secure storage and stewardship of these data over the lifetime of the child may exceed the cost of repeating the genomic testing in the future if the information becomes necessary [17]. The possibility of misusing this information for discriminatory purposes, for example, with regard to employment or insurance, is particularly concerning and must be prevented [3, 4, 118]. The balance struck between the benefits, risks and costs of storing individual data obtained through any genomic newborn screening program is likely to vary among jurisdictions in response to societal and political forces as well as factors like cost and available public health infrastructure.

## Discussion

### Recommendations – further improving public health

Genomic technology provides an opportunity to improve newborn screening by identifying more infants for whom early interventions can prevent serious illnesses, major handicaps or death. However, to be successful, genomic newborn screening must avoid compromising the effectiveness of current screening programs or inadvertently harming children and their families. We, therefore, make eight recommendations regarding the use of genomic technologies for population-based newborn screening.

We consider Recommendations 1-4 to be fundamental and independent of the specific genomic technology used. Recommendations 5-8 are precautionary and relate to the current state of available genomic testing methods. These recommendations should be reconsidered from time to time in the future as our knowledge improves.

*Recommendation 1: Newborn screening by any method, including genomic testing, if adopted as a public health program should be equally available and accessible to every infant born in the jurisdiction.*

The success of current newborn screening programs is largely a reflection of their provision to all, or nearly all, infants. This population-based coverage, which will also be essential for effective genomic newborn screening, requires universal access for all babies. This is a principle of public health as well as a matter of justice.

*Recommendation 2: Interpretation of genomic newborn screening results requires extensive knowledge of the normal (benign) variants, as well as of pathogenic variants, of every gene tested. Genomic newborn screening programs should, therefore, make population-specific allele frequencies of every gene included in the program publicly available in a freely-accessible database. The functional consequences (benign, pathogenic, or undetermined) of each allele should also be made available, along with the evidence supporting functional interpretations.*

Because the diseases that are screened for in newborns are rare (or extremely rare) and because the frequencies of benign polymorphisms, VUS, and disease-causing mutations differ in different ethnic, cultural or geographic populations, sharing information on the pathogenicity of variants internationally in a freely-accessible database will be essential for interpretation of genomic screening results [119, 120]. This must, of course, be done in a way that protects the privacy of individual infants and their families appropriately [121]. Privacy protection is easily managed for common benign variants, which only need to be reported as frequencies (e.g., “327 per 10,000 in Southern Han Chinese populations”) but may be more difficult for disease-causing variants that are so rare that their occurrence in a particular population is limited to one individual or family. In such instances, the individual’s or family’s consent may be necessary for posting the information in a publicly-accessible database, but we believe that most affected families will agree to this to benefit other affected families.

*Recommendation 3: Publicly-funded universal newborn screening by genomic methods should be limited to diseases that can be diagnosed in the newborn period and effectively treated or prevented in childhood.*

In public health programs, limited funding is available and prioritization is required. Unless the screening process for a condition is robust and cost effective, its inclusion in a newborn screening program is more likely to be harmful than beneficial to the performance of the program as a whole. The cost of genome sequencing has fallen dramatically over past 15 years and is likely to continue to fall as a result of ongoing technical advances. Nevertheless, the cost of genome-wide (whole genome or exome) sequencing remains at least 10-100 times greater than any current publicly-funded newborn screening program. Moreover, the sensitivity and specificity of sequencing technology and analytical pipelines have not been shown to be (and are currently probably not) sufficiently high for use in population-based screening [7].

Unless an effective preventative or therapeutic intervention is available to all children who are diagnosed with a condition that is screened for, the program is unlikely to benefit the infants who are being screened in a manner that can be demonstrated to funding agencies. Publicly-funded newborn screening programs, like all public health programs, are held to a high standard of accountability, and if genomic newborn screening compromised the cost-benefit calculation for the screening program as a whole, current newborn screening activities, which have been highly beneficial to many children, could be jeopardized.

*Recommendation 4: If population-based genomic newborn screening is introduced, it should only be offered as part of a comprehensive public health program that includes appropriate confirmatory testing, therapeutic interventions, clinical follow-up, genetic counselling, quality assurance, public and professional education, and governance and oversight.*

As discussed above, the success of current newborn screening programs depends on systematic screening, diagnosis, and management of affected infants through established policies and protocols. Efficient administration and effective governance are also necessary, along with ongoing monitoring and evaluation. Genomic newborn screening would probably be more complex than current screening programs and would, therefore, need to build on the strengths of current programs and operate as a comprehensive system that is available to every infant.

*Recommendation 5: Newborn screening by next-generation sequencing or other genomic methods should only be considered as an add-on to current first-tier screening programs.*

*Recommendation 6: Current newborn screening should not be replaced by next generation sequencing or other genomic methods for any disease unless the genomic technology has been shown to have equal or better sensitivity and specificity for the disease.*

These recommendations are consistent with the Wilson and Jungner criteria (Table 2) and more recent analyses of their application to genome screening at its current state of development for clinical testing [22, 122]. Some conditions for which newborn screening is widely performed cannot be identified effectively by any method of genetic or genomic testing because many cases do *not* have a genetic cause. For example, congenital hypothyroidism may be caused by maternal dietary iodine deficiency or transfer of maternal anti-thyroid antibodies across the placenta. In other circumstances, even though genetic factors usually cause the condition, genetic heterogeneity and complexity make it unlikely that genetic testing will be as sensitive or as specific as current screening methods. Newborn screening for congenital hearing loss by testing otoacoustic emissions provides a clear example. Substituting genomic methods for the methods that are currently used to screen for such conditions would jeopardize the health or development of some children who are identified by current newborn screening programs.

Even for conditions in which genetic heterogeneity and complexity are of less concern, genomic testing may not currently be the most robust method for population-based newborn screening. Bodian and associates studied 1696 infants who had undergone whole genome sequencing and conventional newborn screening in a state-sponsored program [7]. Whole genome sequencing data from these infants were analysed for possible disease-causing variants of 163 genes involved in diseases that either are routinely tested or are being considered for testing in American newborn screening programs. The average infant in this study carried one variant detected by sequencing that was annotated as pathogenic (median = 1, range 0-6). The state newborn screening program identified 4 of 5 infants with a currently targeted disease, while whole genome sequencing identified only 2 of these 5 infants. Among the 27 diseases (associated with 65 genes) tested for in the state newborn screen program, there were fewer false positive results but more results of uncertain clinical significance with whole genome sequencing.

Genomic methods such as next-generation sequencing could, at least in theory, detect some infants with potentially treatable early-onset genetic conditions that are not currently being identified by newborn screening [3–8]. The *addition* of such conditions to the newborn screening panel may be beneficial and cost-effective, but research is required to demonstrate that this is true.

*Recommendation 7: At the present time, our understanding of, and ability to interpret genomic variants does* not *justify use of genome-wide (whole genome or exome) sequencing in population-based newborn screening. Research is needed to demonstrate the clinical utility and cost-effectiveness of genome-wide sequencing and to resolve outstanding health policy and ethical issues before genome-wide sequencing is implemented for newborn screening within a jurisdiction.*

The diploid human genome consists of more than 6,000,000 base pairs of DNA, and every person has millions of differences in comparison to the human reference sequence. Some of these variants are known to be benign, occurring as frequent polymorphisms in healthy individuals. Other variants are known or very likely to cause genetic disease. Many other variants cannot be classified as being benign or disease-causing; genome-wide sequencing identifies many such VUS in every individual [123, 124]. A typical exome from a person who does not have a mendelian disease includes more than 100 novel or rare variants that are predicted to alter protein function [123, 124]. Clinical diagnosis of a genetic disease from genome-wide sequencing data requires recognition of the one or two variants that actually cause the disease in this large background of other variants that are present but have nothing to do with the condition.

As discussed above, interpretation of genomic sequencing results is the biggest practical problem in using this methodology for population screening of newborn infants [91, 93, 94]. The high-throughput sequencing and bioinformatics infrastructure and expertise required to interpret exome or whole genome data from many thousands of infants each year are beyond the capacity of current publicly-funded programs and would be very costly to put into place. Moreover, even when a genomic variant can be interpreted with certainty as pathogenic, predicting the resulting phenotype may be difficult. Different mutations of a single genetic locus can cause different diseases, and identical mutations in different individuals can cause disease manifestations of strikingly different severity.

It is often difficult to obtain the evidence of cost-effectiveness and therapeutic efficacy needed to justify the addition of one condition to the newborn screening panel. The rarity of genetic diseases in infants frequently confounds rigorous cost-benefit analysis and makes randomized controlled trials of the efficacy of therapeutic interventions infeasible. It is hard to imagine how such data could be collected for all genetic diseases that might be identified by genome-wide sequencing, and obtaining these data just for the conditions covered by a large gene panel would pose immense problems. Thorough assessment of the success of initial efforts at population-based genomic newborn sequencing will certainly be necessary.

*Recommendation 8: At the present time, our understanding of, and ability to interpret genomic variants does* not *justify sequencing large multigene (physical or bioinformatic) panels for population-based newborn screening. Research is needed to demonstrate the clinical utility and cost-effectiveness of sequencing large multigene panels for population-based newborn screening and to resolve outstanding health policy and ethical issues before the use of large sequencing panels is implemented for newborn screening within a jurisdiction.*

Most current suggestions for expanding newborn screening through sequencing of disease genes propose to do so by using targeted panels [17, 125]. Interpretation of variants found on gene panels with respect to pathogenicity presents the same difficulties as interpretation of the variants found by sequencing individual genes but multiplies these problems by as many genes as there are on the panel. Although sequencing a small panel of genes is much less likely than genome-wide sequencing to produce incidental findings or VUS, these issues are unlikely to be completely resolved, and the advantages are lost as the gene panel becomes larger [4, 99]. The more genes included in the panel, the larger the proportion of variants for which the association with disease is uncertain. The penetrance, variability and natural history of disease caused by particular mutations become more uncertain as the number of genes on a panel increases, and the frequency and distribution of benign polymorphisms in various populations is more often unknown.

In any case, we do not currently know enough about the pathogenic consequences of the full population spectrum of variants for any disease gene, and much less for a panel of disease genes, to justify the use of such sequencing as a primary method of newborn screening. More research in this area is needed.

## Conclusions

The inclusion of genomic sequencing in newborn screening presents a major opportunity to detect and effectively treat or prevent many more serious child health conditions than is possible today. However, before genomic sequencing can be implemented in a newborn screening program, clinical utility and cost-effectiveness must be demonstrated [37, 110, 111]. A key issue is the need to improve the interpretation of genomic data to permit robust recognition of both disease-causing and benign variants of all genes screened in every child in the population. In addition, a consensus needs to be developed within each jurisdiction on ethical and policy controversies such as the disclosure of genomic VUS and incidental findings to families, ownership of the data, and appropriate data storage and sharing. Revision of our recommendations will be needed as more information becomes available.

The best interests of children should remain the guiding principle in newborn screening and the basis for decisions regarding the implementation of genomic newborn screening.

## Abbreviations

PKU: Phenylketonuria
VUS: Variants of uncertain significance
